# Supplementary material for: Magnetic origami creates high performance micro devices
Source: Nat Commun. 2019 Jul 8;10:3013. doi: 10.1038/s41467-019-10947-x (PMC6614421; doi:10.1038/s41467-019-10947-x)
Supplement: Supplementary file 1 — Supplementary Information [file 41467_2019_10947_MOESM1_ESM.pdf]

Supplementary information

Magnetic origami creates high performance micro devices

Gabler et al.

Supplementary Figures

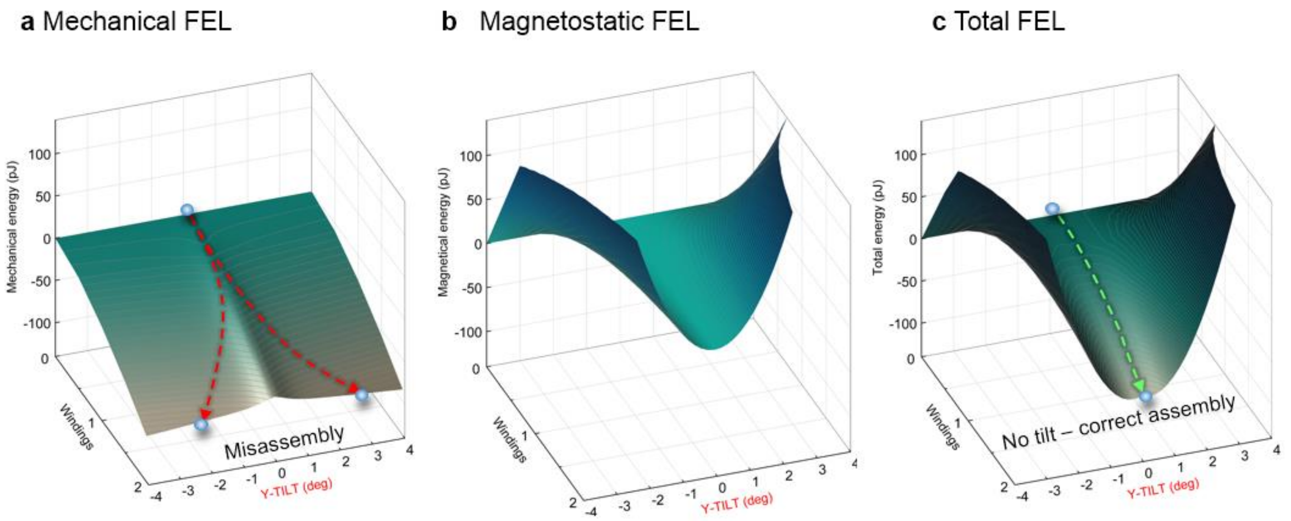

Supplementary Figure 1 | Effective elimination of y-tilt in an external axial magnetic field
a, Mechanical strain energy versus tilting and number of windings. **b,** Magnetostatic energy versus tilting and number of windings for trapezoidal and rectangular designs. **c,** Sum of the mechanical strain energy (**a**) and the magnetostatic energy (**b**).

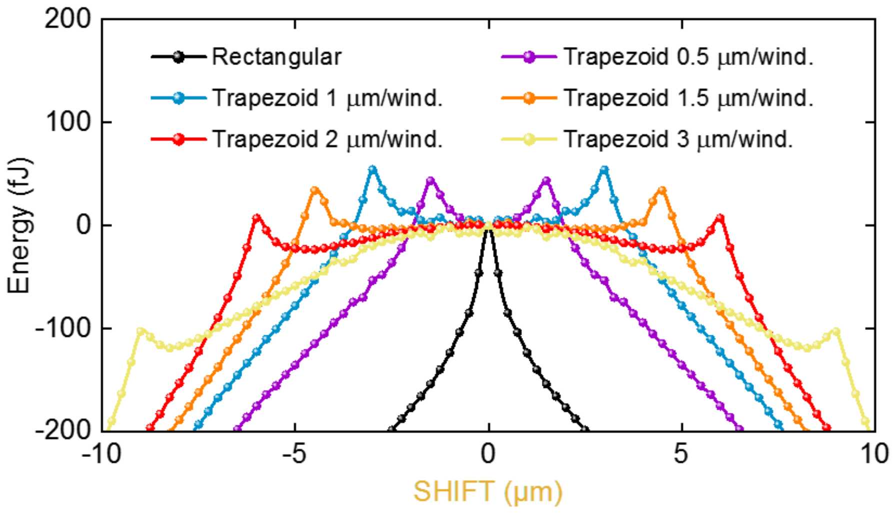

Supplementary Figure 2 | Magnetostatic energy profile as a function of shift
Magnetostatic energy defined for rectangular and trapezoidal designs with different pitch at three windings.

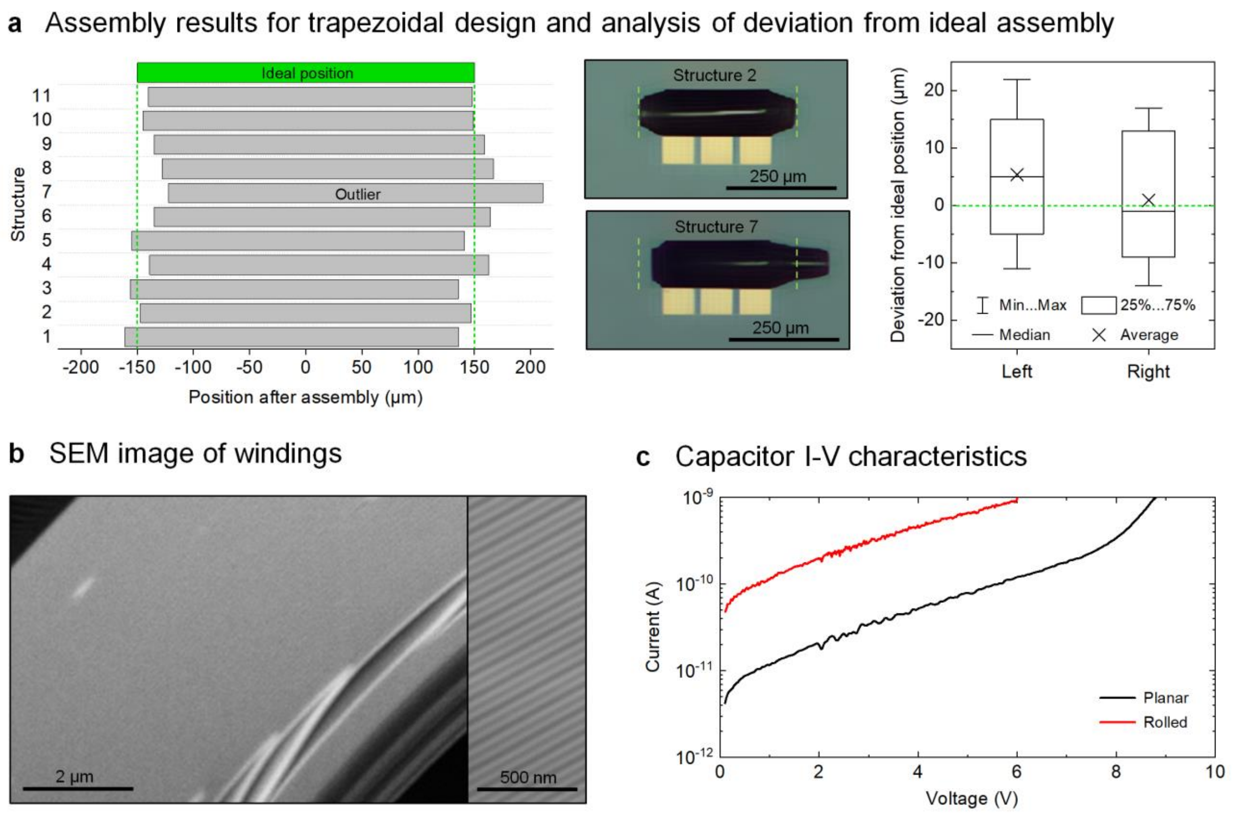

Supplementary Figure 3 | Statistical deviation and further details of capacitors

Given data are for capacitors fabricated by axial magnetic field assisted assembly. **a,** Assembly results of trapezoidal design with a pitch of 1 µm/winding, optical microscopy image of two exemplary structures after assembly and statistical analysis of deviation from ideal position. **b,** Scanning electron microscopy image of capacitor cross section showing about 50 windings. **c,** Current-voltage characteristics of capacitors with 15 nm Al_2O_3 thickness in planar and rolled state.

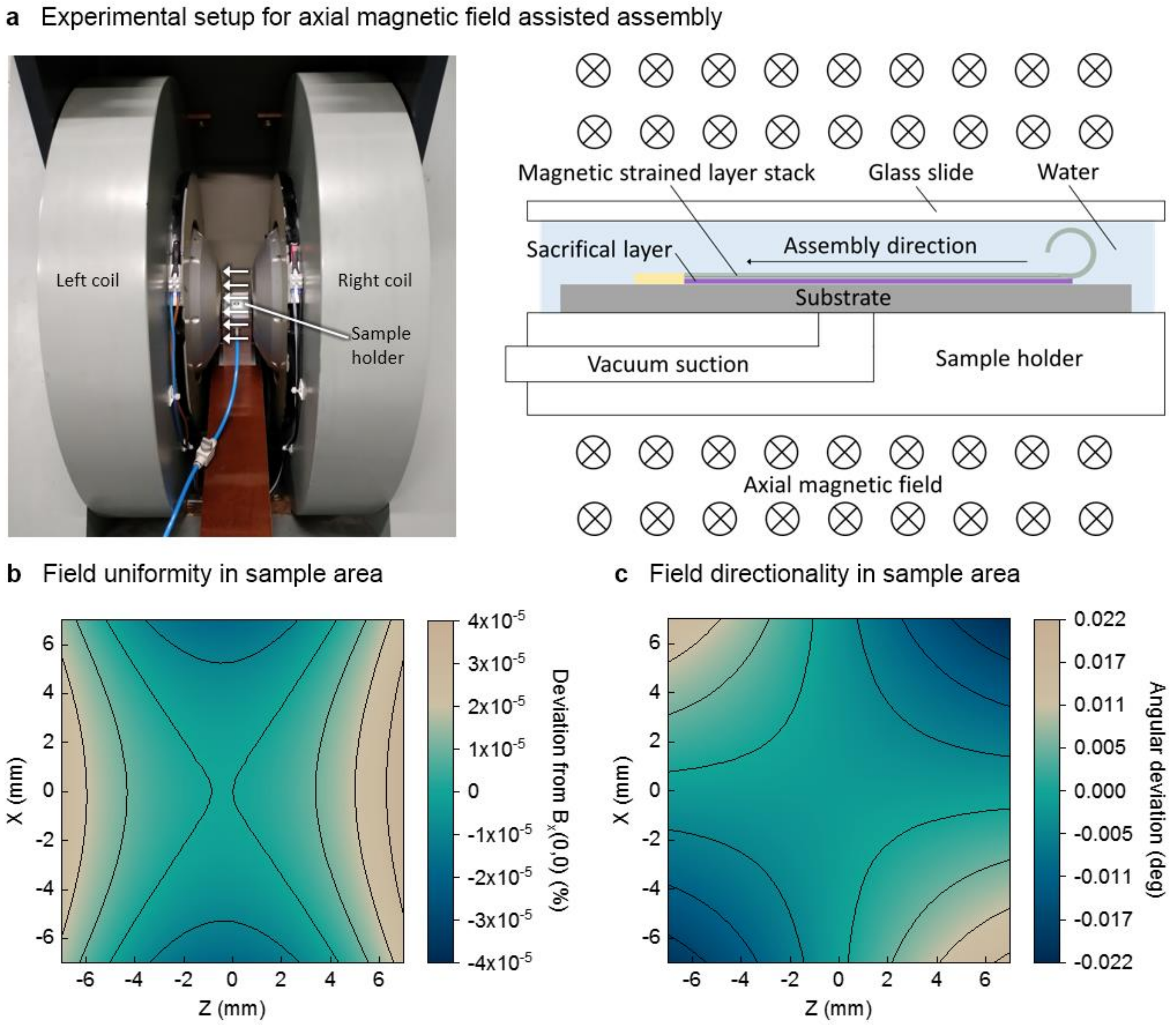

Supplementary Figure 4 | Experimental setup for axial magnetic field assisted assembly
a, Photo of dipolar electromagnet and schematic of assembly in axial magnetic field. **b,** Field uniformity in 14 x 14 mm² sample area. **c,** Field directionality in 14 x 14 mm² sample area.

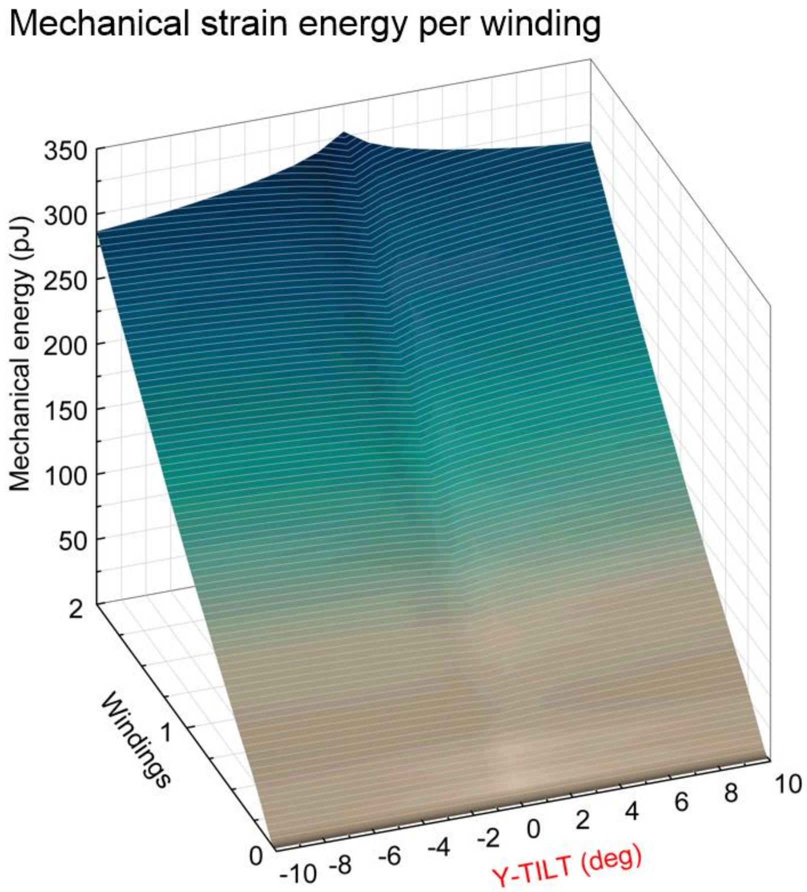

Supplementary Figure 5 | Mechanical strain energy surface for tilt and windings
Calculated FES that arises from the winding process of the metal film without intrinsic stress. Lower energy shoulders occur in the tilted case leading to larger diameter windings and, therefore, tending to a misalignment.

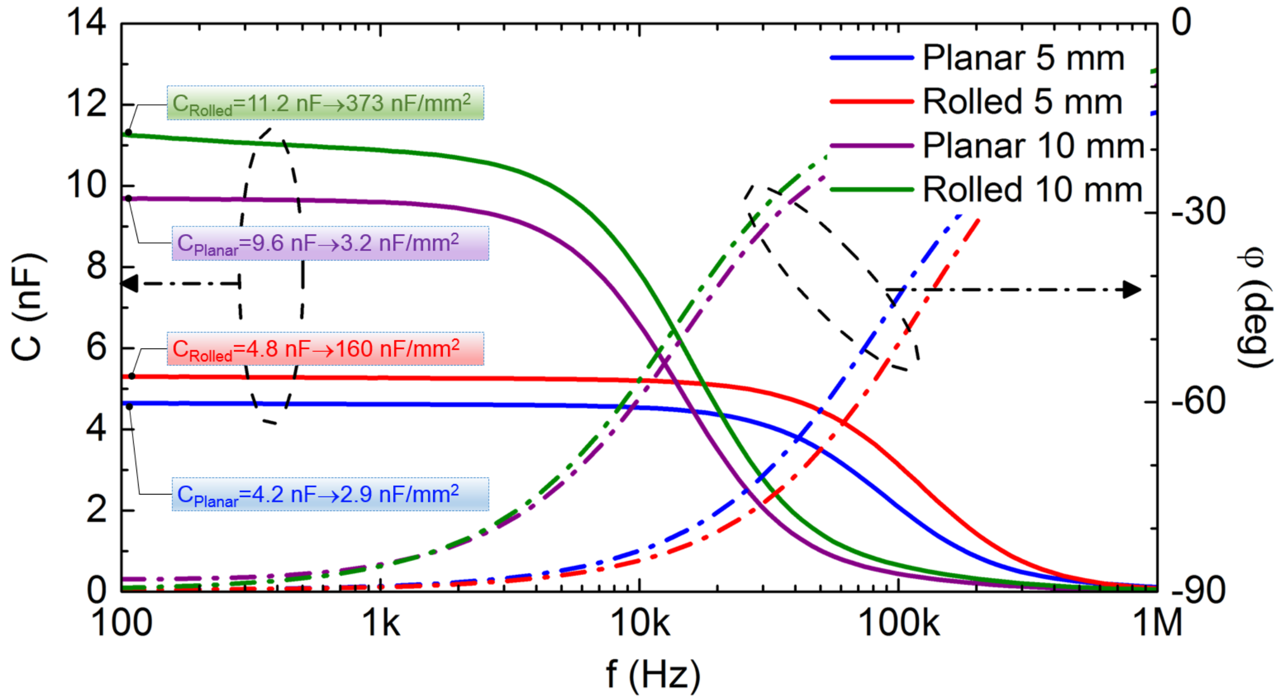

Supplementary Figure 6 | Electric performance of dry planar and assembled capacitors
Data given for 5 mm and 10 mm rolling length of the assembled nanomembranes revealing a clear difference in capacitance and capacitance density for planar and rolled cases.

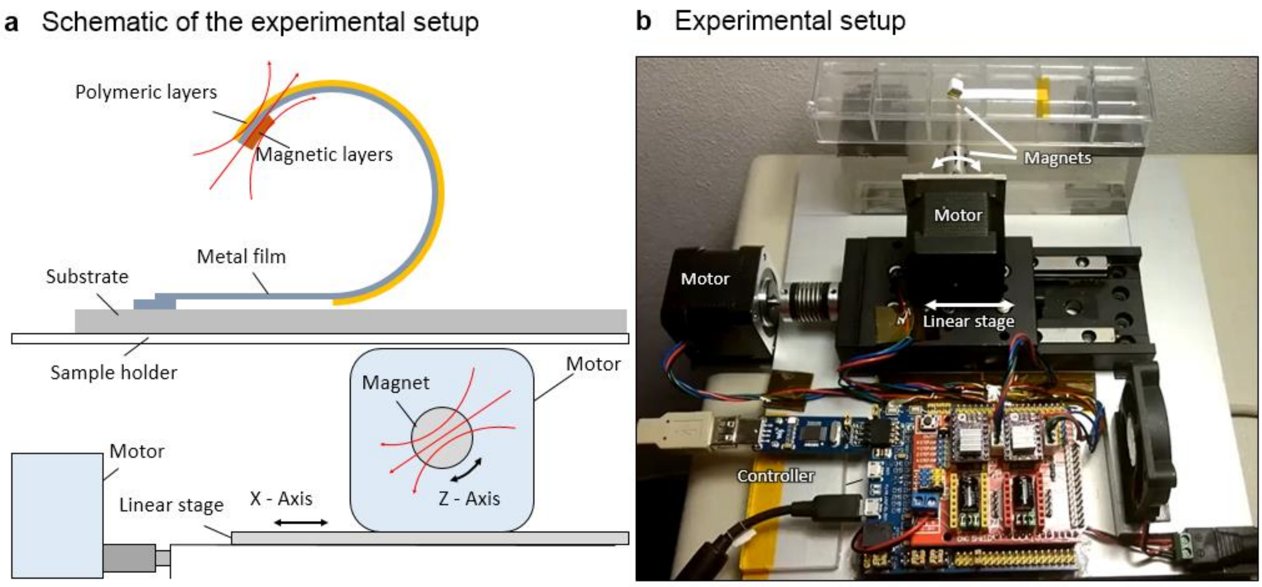

Supplementary Figure 7 | Set-up for the radial magnetic field assisted assembly
a, Schematic representation of the experimental setup producing rotating magnetic field. **b,** Arduino based experimental setup and a macroscopic experiment showing assembly of a paper stripe into a "Swiss roll" with the help of a rolling magnet in a rotating magnetic field.

Supplementary Notes

Supplementary Note 1 Elimination of y-tilt and alignment along the field direction

We calculated two free energy surfaces (FES) for mechanical strain energy of the "Swiss-roll" architecture (Supplementary Figure 1a) and magnetostatic energy (Supplementary Figure 1b) revealing three dimensionally complex profiles. Appearance of the Y (TILT) can be seen in Supplementary Figure 1a as left and right shoulders with negative slope compared to the zero tilt case in the middle of the graph. Such a FES profile appears due to a preferential helical assembly (see Fig. 3a bottom inset) of the thin film rather than a multiwinding "Swiss-roll". This happens due to the larger diameters of the outer windings that cannot fully relax the strain energy when all of the windings are aligned during the self-assembly process at zero tilt angle. Thus, the assembly with a tilt is preferred and leads to a helical shape where each of the windings possess similar diameters and as a result more relaxed strain energy. The magnetostatic FES (Supplementary Figure 1b) demonstrates an increase in energy for left and right tilts of the soft ferromagnetic "Swiss-roll" structure in external magnetic field. The sum of both of these energies results in a complex FES (Supplementary Figure 1c) that show the lower energy state, which is always at zero tilt during

relaxation of the intrinsic strain energy and self-assembly of the structure. This behavior however does not automatically remove the SHIFT of windings that can happen even when the whole structure is aligned along the field direction. This point is discussed in the main text of the manuscript.

Supplementary Note 2 Magnetostatic energy as a function of shift

The simulation of the magnetostatic FES revealed substantially different shapes depending on the initial planar design of the nanomembrane. A change from rectangular to trapezoidal design results in the formation of energy plateaus with new local minima. However, the specific shape of these plateaus depends on the trapezoid pitch (Supplementary Figure 2). Ideally, the FES should exhibit a concave plateau around zero SHIFT confined by preferably high-energy peaks. Furthermore, the plateau should be not too narrow to confine the SHIFT values during the formation of the first two windings within the emerging energy valley. For the given simulation geometry (300 µm width, 60 µm diameter, 15 nm layer thickness and 30 nm layer spacing) a trapezoid pitch between 0.5 and 1 µm/winding turns out to be optimal. It can be advantageous to choose a larger pitch in the beginning and reduce it along the assembly path.

Supplementary Note 3 Further details of axial magnetic field assisted assembled capacitors

Supplementary Figure 3a shows the assembly results of 10 mm long nanomembranes with trapezoidal design having a pitch of 1 µm/winding. The width of the stripes was 300 µm at the widest position and 200 µm at the bottom. Out of 11 assembled structures, 10 showed quasi ideal assembly with a total SHIFT below 25 µm after about 50 windings (Supplementary Figure 3b). This corresponds to a yield of about 90 %. Exemplary I-V characteristics of assembled capacitors with 15 nm Al_2O_3 dielectric are shown in Supplementary Figure 3c. During measurement the leakage current was limited to 1 nA in order to prevent breakdown. The capacitors reach 8.8 V in planar and 6 V in rolled state. The decrease in voltage can be contributed to decreased insulation resistance after the assembly process because the bottom oxide layer gets in contact with the top metal layer increasing the effective conductor-insulator interface.

Supplementary Note 4 Setup for axial magnetic field assisted self-assembly of nanomembranes

The sample is placed inside a PTFE sample holder and covered with a glass slide at a vertical distance of about 1 mm to avoid water turbulence. The sample holder is placed in central position inside a water cooled dipole electromagnet with 50 mm pole gap and 115 mm pole face diameter (Supplementary Figure 4a). Supplementary Figure 4b shows the deviation of the magnetic field's z-component inside the 14 x 14 mm² sample area with respect to the central value $B_z(0,0)$ at 10 kOe magnetic field. The corresponding angular deviation of the magnetic field $\vec{B}$ from the z-axis is shown in Supplementary Figure 4c. As shown in the figures, the field is uniform and unidirectional. As a consequence, misalignment caused by tilted field lines or forces caused by any field gradient are neglected.

Supplementary Note 5 Mechanical energy of the system (radial case)

Supplementary Figure 5 shows calculated FES arising from the winding process of the metal film without intrinsic stress. This simulation shows similar behaviour as in Supplementary Figure 1a where left and right shoulders have negative slope compared to the zero tilt case and a positive slope. Lower energy shoulders occur in the tilted case leading to larger diameter windings. The potential energy difference in the mechanical FES due to tilt is an order of magnitude lower than the height of the magnetostatic potential energy barriers between local minima shown in Fig. 5c, which effectively prohibits misalignment due to this DoF.

Supplementary Note 6 Electric characterisation of capacitor structures (radial case)

We characterize capacitor structures before and after the assembly process revealing an enhancement of capacitance (Supplementary Figure 6), which reaches 4.8 nF and 11.2 nF compared to their planar counterparts of 4.2 nF and 9.6 nF, respectively, measured at 100 Hz. The assembly process reduces the footprint area of each structure, which simultaneously enhances the capacitance density from 2.9 nF/mm^2 and 3.2 nF/mm^2 to 160 nF/mm^2 and 373 nF/mm^2, respectively, for shorter and longer

structures. These capacitances are measured in dry conditions when the devices were removed from the rolling solution, washed and dried.
